# A Cat Skeleton from the Balatlar Church Excavation, Sinop, Turkey

**DOI:** 10.3390/ani11020288

**Published:** 2021-01-23

**Authors:** Vedat Onar, Gülgün Köroğlu, Altan Armutak, Öğül Emre Öncü, Abu B. Siddiq, Aleksander Chrószcz

**Affiliations:** 1 Osteoarchaeology Practice and Research Centre and Department of Anatomy, Istanbul University-Cerrahpaşa, 34320 Istanbul, Turkey; 2 Division of the Art History, Bomonti Campus, Mimar Sinan Fine Art University, 34380 Istanbul, Turkey; gulgun.koroglu.27@hotmail.com; 3 Osteoarchaeology Practice and Research Centre and Department of Veterinary History and Deontology, Istanbul University-Cerrahpaşa, 34320 Istanbul, Turkey; armutak@istanbul.edu.tr; 4 Istanbul Archaeological Museum, Osman Hamdi Bey Street, 34122 Istanbul, Turkey; oeoncu@gmail.com; 5 Department of Anthropology, Mardin Artuklu University, 47200 Mardin, Turkey; abubakarsiddiq@artuklu.edu.tr; 6 Department of Biostructure and Animal Physiology, Wrocław University of Environmental and Life Sciences, Kożuchowska 1, 51-631 Wrocław, Poland; aleksander.chroszcz@upwr.edu.pl

**Keywords:** cat burial, byzantine pet, balatlar church excavation, *Felis catus*, Turkey

## Abstract

**Simple Summary:**

A cat skeleton was unearthed during the 2015 excavation season at the Early Byzantine Balatlar Church complex, by the northeastern Black Sea coast of Turkey. The cat was buried with a human individual. The inhumation was dated back to the period between the end of the 6th century AD and the first half of the 7th century AD. The sex of the human individual remains unknown and the cat has been identified as a female house cat. The skeletal remains in the region of the abdominal cavity, occupied by the stomach in living animal, revealed the remains of a rodent and a house sparrow, eaten only recently prior to cat’s death. This can be interpreted as an indirect proof of the cat’s role as an efficient pest controller, alongside that of being a pet animal. Presenting the zooarchaeological and archaeological evidence, we argue that the Balatlar cat and her possible owner in the “2015-Grave-14” burial chamber demonstrate the most significant direct archaeological evidence of a cat–human relationship in the Byzantine world so far.

**Abstract:**

In the 2015 excavation season, an east–west oriented burial (2015-Grave-14) built with large dimension stone blocks was unearthed on the south edge of “Area IVi” at the Balatlar Church in Sinop, on the northeastern Black Sea coast of Turkey. In this grave, which is dated between the end of the 6th century AD and the first half of the 7th century AD, a human skeleton was found with the head to the west and a cat skeleton was carefully placed next to the right femur. This study on the burial and the cat skeleton within it shows that, compared to the Roman period, the status of cats reached a higher level during the Byzantine period. It was found that alongside of being a pet, the Balatlar cat was a young healthy female individual that instinctively hunted rodents and birds, given that the remains of a rat and a sparrow were found in the region of the abdominal cavity, corresponding with the stomach location in the living animal. The grave presents the most significant direct archaeological evidence of a pet–human bond recorded at any Byzantine site so far.

## 1. Introduction

Human–cat relationships in the modern world are claimed to have developed and reached a significant level through a long historical process. However, there is still scare archaeological evidence of the domestication of these animals [1]. Egypt is considered as the center from which the domestic cat spread around the Old World [2]. In particular, ancient DNA analyses have proven different routes of domestic cat migration, from Egypt to different other parts of the Ancient World [3]. In this way, the variety and wider distribution of domestic cat populations originated and increased [1]. This process in the Old World gained momentum during the classical period and it is argued that both maritime and terrestrial trade networks played crucial roles in the spread of cats [3]. The transmission of the domestic cat from Egypt to Europe also occurred through various routes. The earliest example of such spread was found via evidence in the form of 5th–4th century BC Greek art works in the southern region of the Italian peninsula [4]. 

In Turkey (ancient Anatolia), domestic cats are known to have existed at least from the Roman period [5]. Roman poet Ovid’s reference to the sanctity of the Egyptian cat can be attributed to the evidence that Romans were familiar with domestic cats from 100 BC [6]. Yet, the term “domestic cat” or “cat” was rarely mentioned in Roman literary texts, perhaps because they were described as “pest controllers” for killing rats and mice in households [5]. Cats’ domestication process has led them to play an important sanitary role in human settlements [1]. Simultaneously, the role and the importance of the cat in household probably did not significantly increase before the transition from the classical to medieval period [7]. Arguably because of this reason, the domestic cat was not mentioned in Roman literary sources [8], including the great agricultural lore Geoponica (6th century AD), or in the works of the prominent Roman agriculture writer Columella (1st century AD) [5]. However, although such negligence was present in terms of highlighting the contributions of house cats in human society, this perhaps did not prevent them from being a favorite pet animal. 

The domestication of cats marked the beginning of the acceptance of cats as pet animals [1]. As pets, they gained the scope to enter the inner sphere of the human world. Among medieval societies, cats were generally seen as a symbol of possessing luxurious goods—in addition to emphasizing the owners’ desire to express their high social status and exhibit their material assets—the way cats were kept was seen as an identity defining factor of their owners [9]. This was a very different situation than keeping a cat as a rodent controller in the household. The task of pest control was at the forefront of the cat domestication process, which was associated with a commensal relationship between humans and cats [10]. Before the widespread increase in the perceived value of cats, both the Greeks and Romans preferred snakes and weasels for killing rodents and pest control [11]. However, given the fact that they are cleaner animals for the tasks—as well as good companions at home—instead of snakes and weasels, domestic cats started to be preferred throughout the 2nd–5th century AD [6]. 

According to the Roman author Pliny the Elder [12], domestic cats replaced mustelids in the early Christian era [10]. It is also suggested by ancient writers, however, that the terms weasels and cats were used interchangeably, without making any distinction [13]. It is argued that the lack of popularity resulted in the domestic cat having not yet received a standard name at that point in time [14]. Nevertheless, from 200 AD the words “catus” and “catta” were commonly used for describing the cats [5]. 

The only thing that cannot be determined from the cat remains found in Roman archaeological sites was the actual status of these animals and the question of whether they were used as “pest controllers” or “pets” at these settlements [15]. Zooarchaeological evidence has indicated the existence of human–cat relationships for thousands of years [16,17,18]. Yet, due to the scarcity of cat remains in archaeological records, current hypotheses for early cat domestication are based on only a few zooarchaeological case studies [3]. The rarity of cat remains in the Graeco–Roman archaeological context [19] also restricts our knowledge about the role and functions of cats, as well as their status of being domestic or wild cats. It appears that there were no early finds of domestic cats in Anatolia [5]. Considering the small number of cat remains obtained from only a few Roman sites, including Didim [20], Pergamon [21], Lidar Höyük [22], Pessinus [23], Troy [24] and Sagalassos [5], it can be argued that domestic cats were brought to Anatolia at least during the Roman period [5]. 

On the other hand, although some argue the dominance of the negative image of cats in Byzantine records [25], it should be questioned whether this existed in the early Byzantine time. Besides of being pet animals, cats were also used in the Byzantine world to keep rodents under control [26]. Despite the scarcity of cat remains at the Roman sites in Anatolia, a very rich assemblage of cat remains from the Yenikapı Metro and Marmaray excavation demonstrated that cats played a remarkable role in the life of the Byzantine capital Constantinople [27,28,29,30]. The Yenikapı cat assemblage belongs to the Early Byzantine (4th–7th century AD) to Late Byzantine period (15th century AD) [28], and when their quantities are compared, it appears that to date, no other Byzantine site has yielded such a large assemblage of cat remains. The Yenikapı remains also showed that, besides of being pets, cats continued to play a significant part in the urban life of Constantinople. On the other hand, the fact that cats were among the favorite animals of the Byzantine empress Zoë (1028–1050) [25], also demonstrates the higher status of cats in Byzantine society. In this regard, the Balatlar cat presents the most significant direct archaeological evidence of a cat–human bond found at any Byzantine site so far. 

## 2. Materials and Methods 

### 2.1. Achaeological Site 

**Figure 1:**
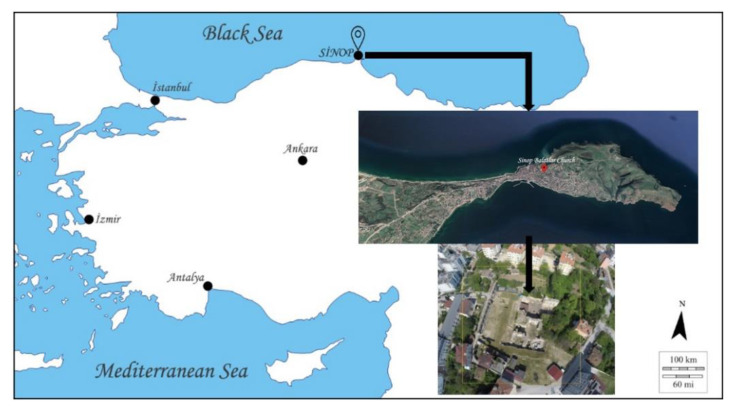
Location and overview plain of the Balatlar Group Complex in Sinop (Base & above: free maps, “http://www.d-maps.com” & Google Earth; below: Balatlar Excavation Archive).

**Figure 2:**
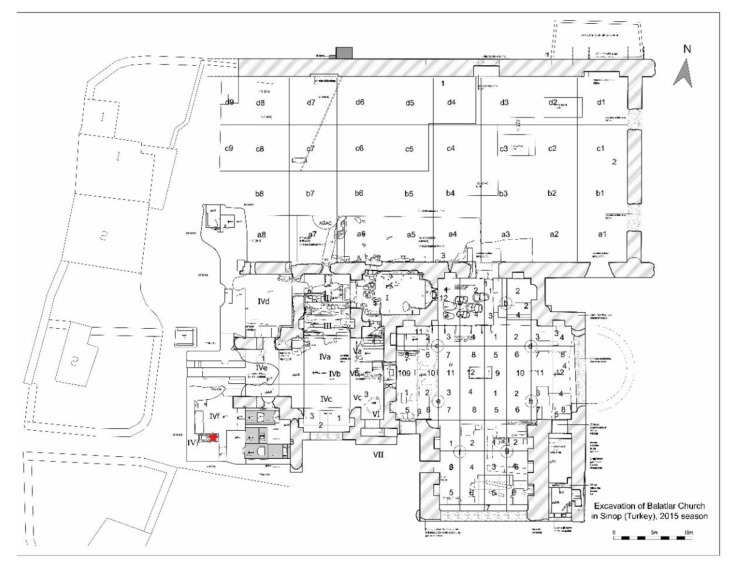
Areas under 2015 excavation at the Balatlar Church complex: the red star indicates “2015-Grave 14” burial chamber.

**Figure 3:**
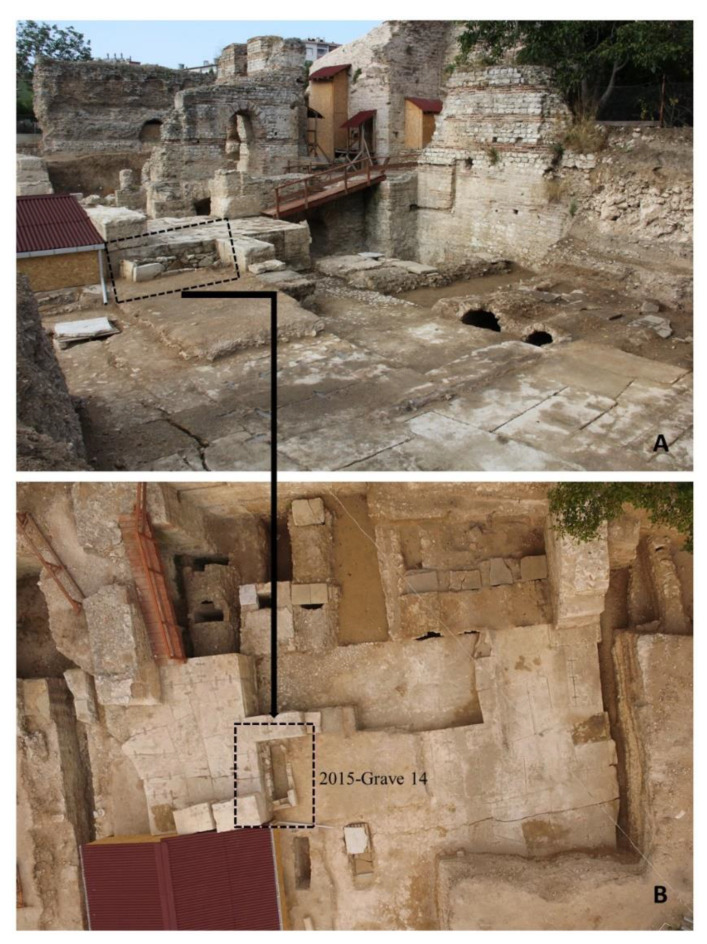
Close view of the “2015-Grave-14” burial chamber at the Balatlar Church complex.

The Balatlar Church complex lies in the ancient Paphlagonia region and at the heart of the present provincial city of Sinop, at 42°01′34″ north latitude and 35°09′25″ east longitude, where the Boztepe peninsula connects to the mainland [31] on the northernmost edge of the Turkish Black Sea coast (Figure 1). Archaeological excavations at this building complex began in 2010 under the direction of Gülgün Köroğlu and the site gained its official name as the “Balatlar Church”. It was found that the original purpose of this building complex was as an imperial bath during the Late Roman period, later it was converted into a church and was spread over a wider area [31]. The complex is comprised of building remains of different architectural styles. Although known as the Byzantine “church” among the public statements and in archaeological studies, the site revealed several archaeological layers, including the evidence of occupation during the Late Hellenistic, Roman, Byzantine, Seljuk and Ottoman periods [32,33]. The site was an active royal bath complex during 3rd–4th century AD [32]. Following the acceptance of Christianity and its affirmation as the official estate religion, the caldarium (heat section) of the building was converted into a church during the mid-4th or 5th century AD [32,34]. In the 7th century, a small chapel with a single nave was added to its south cross arm. From the 12th or 13th century until the first quarter of the 20th century, the northeast corner room of the tepidarium (tepidity) of the bathhouse (Room No. I) continued to be used as a church (Figure 2 and Figure 3). 

**Figure 4:**
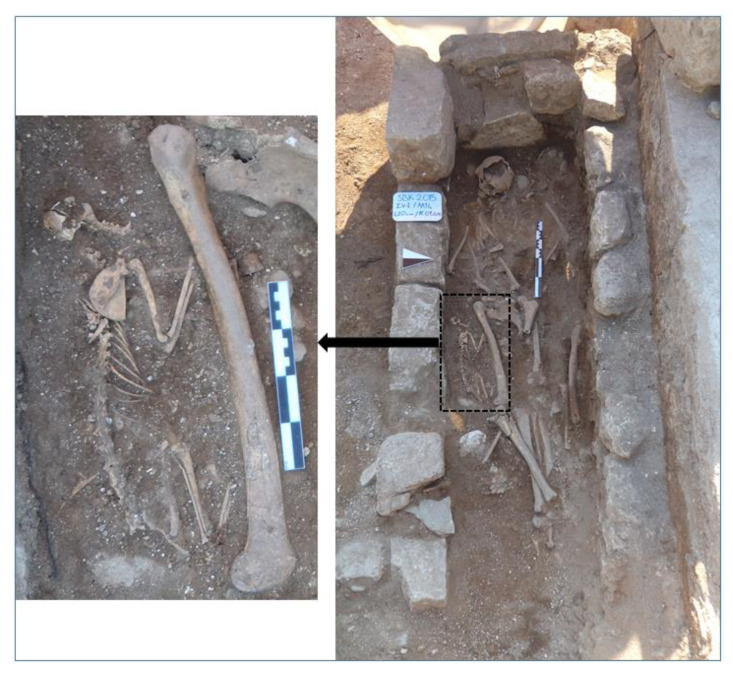
In situ view of the cat skeleton (Balatlar cat) at the “2015-Grave 14” chamber.

The floor of the church, and especially its surroundings, were used as a cemetery [34], and many of the burials belonging to its early period were unearthed in recent excavations [32]. One of these burials was a burial chamber from “Area IVi” where the cat was buried with a human individual (Figure 4). To construct a comparative dating of this “2015-burial chamber 14” of Area IVi, the burial remains of “Area IVf” to the east were used as a benchmark. The burial chamber of IVf was dated back to between the end of the 6th century AD and the first half of the 7th century AD. Located in the same cultural layer and adjacent to the burial chamber of IVf, it has been suggested that the burial chamber of IVi also belonged to the same period. Beyond being a pet animal with burial status, the cat skeleton of this grave was examined with consideration of an indicator for human–pet animal relationships in the Byzantine world. 

### 2.2. Zooarchaeological Evidence 

A cat skeleton, associated with a human skeleton in the “2015-grave 14” from Area IVi of the Balatlar Church complex, was examined in this study. The cat was carefully placed next to the right femur the human individual, who was the last person buried in the grave chamber. The cat and human skeleton were observed to be synchronous, given that they were the only ones placed in the same depositional layer, which meant they were separated from lower human remains by a distinct soil layer. The sex of the human individual remains unidentified, but is expected to be determined by future studies. The skull of the cat was found to be fragmented, possibly due to the collapse of the chamber. Except the fragmented skull, osteometric measurements were taken from the mandiblae, scapula, humerus, radius, ulna, coxae, femur, tibia, fibula, talus, calcaneus, metapodiums, atlas, axis and sacrum of the skeleton. 

### 2.3. Calculation of Domestic Status and Morphology 

Basic standards of the skeletal measurements were followed from von den Driesch (1976) [35], von den Driesch and Boessneck (1983) [36], Kratochvil (1973, 1976) [37,38], Teichert (1978) [39] and Guintard and Arnaud (2003) [40]. In this way, it was possible to compare the Balatlar cat with cat measurements found in different periods and localities, but close to each other. In addition to these archaeological examples, the cat was also compared with the measurements of an Angora cat in our laboratory, in order to make a comparison with a modern sample. The comparison and evaluation of the measurements revealed possible morphological differences of the Balatlar cat from those presented in the wild cat (*Felis silvestris*) and modern domestic cat (*Felis catus*). 

A Z-score calculation was further performed to compare raw morphometric data obtained from the Balatlar cat skeleton with those of the domestic and wild samples. Thus, with the help of the obtained Z score, it was possible to determine which direction (positive or negative) and by how many units the Balatlar cat deviated from the average mean of the domestic and wild cat data. 

### 2.4. Osteometric Measurements 

All morphometric measurements were taken using an electronic slide-caliper and were repeated three times to estimate the mean value. The detalied description of this prcedure is presented in Table 1. The results were compared with the accessible literature and discussed. 

In pet animals, particular index calculations are made using morphometric measurements of the long bones of the front and hind legs. One of these indices, is the slenderness index which is accepted as an indicator of the strength of a particular animal individual and is used to classify whether the front and hind limbs of the individual are thin or thick [41,42,43]. In this study, index calculations were made using measurements of the humerus, radius, femur and tibia of the cat. In this way, the visual morphology of the Balatlar cat was evaluated by comparing these data with the cat indices obtained from different archaeological sites and modern samples. 

Calculated indices: 

Humerus index = SD*100/GL. 

Radius index = SD*100/GL. 

Femur index = SD*100/GL. 

Tibia index = SD*100/GL. 

### 2.5. Animal Age Estimation 

On the basis of time indices for the epiphyseal fusion of the long bones, the animal age was estimated. The dentition status was not evaluated. The strong fragmentation of the cranial skeleton and teeth did not allow for more advanced studies. The number of alveoli suggested that the dental formula for both dental arches was normal and all teeth had erupted. 

### 2.6. Animal Sex Estimation 

Animal sex was estimated on the basis of the existence of the os penis. The presence of this bony structure is typical for all Carnivora. 

## 3. Results 

In the second intermediate period of the 2015 excavation season at the Balatlar Church complex, a grave was unearthed from the end of the bosage surface stone blocks of Trench IVi. The grave was placed in the east direction, covered with large block stones, and associated with an earlier occupational period of the site (Figure 3). When the grave was cleaned and opened, a human skeleton was found lying with the head towards the west and feet towards the east. The cat was placed next to the right femur of this human individual, who was the last person buried in the grave chamber (Figure 4). Although there were multiple burials in the chamber, it was observed that the cat and human skeleton were placed in the upper-most layer, being completely separated from the lower burials by a distinct soil layer. 

The cat skeleton, laid on its left side, was also carefully placed in the east–west direction following the human skeleton. Considering the times of its epiphyseal closure (fusion) of different skeletal parts, it was found that the cat was a young female individual (Table 2). In particular, the absence of the os penis and the fact that the long bones were not large and heavy, but smaller and delicate, directed the determination of the individual to be a young female cat. Both the general morphology of the bones and morphometric examination of the skeletal remains indicated the remains to belong to a domestic cat. Moreover, considering the fact that it was carefully placed in a human grave, in proximity to the human individual, the cat appeared to be a pet animal. 

**Figure 5:**
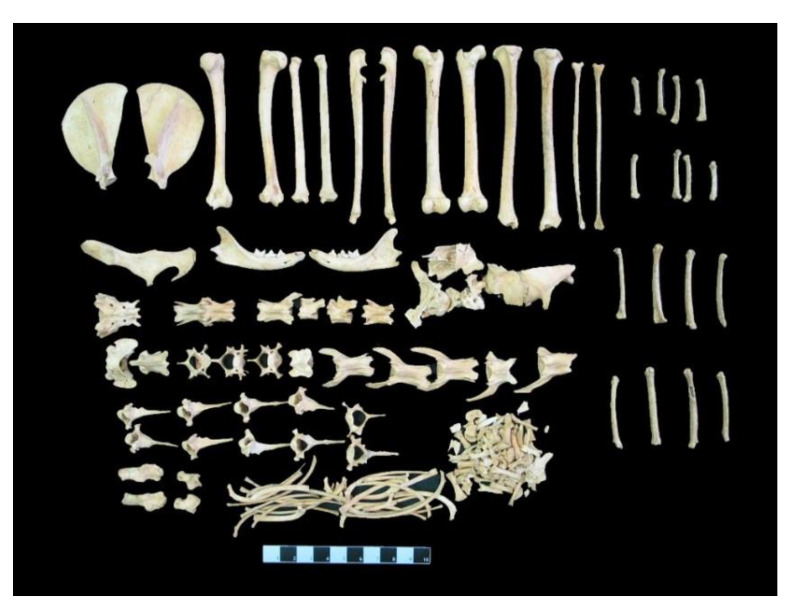
The skeletal remains of the Balatlar cat.

Almost all skeletal elements of the cat were preserved (Figure 5). However, the skull was smashed into pieces, apparently because of the collapse of the grave and therefore it is not available for further studies. Therefore, except for the cranial skeleton, it was possible to take morphometric measurements from all other skeletal parts (i.e., mandible, scapula, humerus, radius, ulna, ossa coxae, femur, tibia, fibula, talus, calcaneus, metapodiums, atlas, axis and os sacrum). The obtained morphometric measurements and their comparison with the measurements of domestic and wild cats (given as mandible measurements only) from different archaeological sites are presented in the tables (Table 3, Table 4, Table 5, Table 6, Table 7, Table 8, Table 9, Table 10, Table 11, Table 12 and Table 13). Moreover, the Angora cat data were used in morphometric comparisons with modern cats. 

To make the comparison more reliable, the bone measurements of the Balatlar cat were further compared with the raw measurements of the wild cat and domestic cat by using Z-scores. In particular, the greatest length (GL) values of the bones, which are thought to be more effective in predicting visual morphological characters of a species, were used for Z-score calculations. In this way, together with the raw morphometric values, the Z-score presented how many units of the GL value deviated in the negative or positive direction from the average mean of the domestic and wild cat data. 

The morphometric measurements of the skeletal remains of the Balatlar cat did not suggest it was a large sized cat. Instead, with it showing characteristics of a young female individual, the animal appeared to be a regular sized house cat. 

The thoracic and pelvic limbs were found to be thin and slender according to the calculations obtained from the long bone indices (humerus, radius, femur and tibia indices) (Table 14). Being a young individual, in addition to perhaps the sex of the animal could both have had an effect on this. No pathological marks or health problems were observed during the careful macroscopic examination on the skeletal remains. This all suggested the individual to be a regular-sized normal healthy female house cat. 

**Figure 6:**
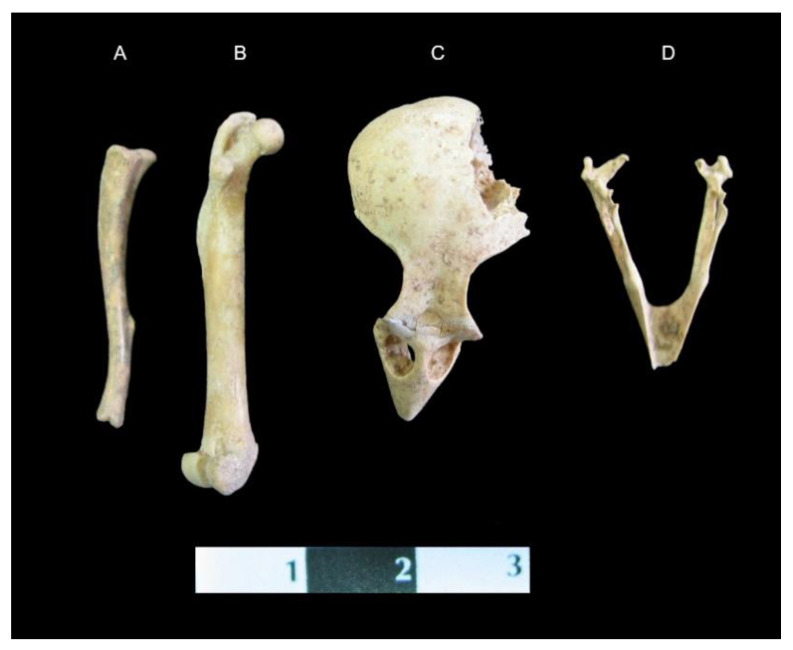
Skeletal remains recorded from the stomach of the Balatlar cat: **A**. Tibia of *Rattus rattus*; **B**. Femur of *Rattus rattus*; **C**. Skull of house sparrow (*Passer domesticus*); **D**. Mandible of house sparrow.

An astonishing finding is the presence of the other animals’ remains, found together with those of the cat. The cat skeleton was unearthed in the anatomical composition as an articulated skeleton. The area corresponding with the abdominal organs location was occupied by the remains of a bird and a rodent, in the topographical location of the stomach in a living animal (see Figure 6). The lack of digestive marks (typical of biting or chewing) on the bones suggests that both skeletons were from the cat’s prey. Archaeozological analysis proved that the mentioned bone fragments belonged to the house sparrow (*Passer domesticus*) and the house rat (*Rattus rattus*). Both animals could have been captured by the cat shortly before its death. It seems to be impossible that the cat could have swallowed both animals whole; therefore, the evidence of them being within the grave is a little confusing. Maybe both forms of prey were buried together with the cat and its owner because of his social status, the unknown importance of the animals, or even as part of a form of ritual. On the other hand, the location of these findings in the context of the topographical anatomy of the cat—i.e., their presence in an area normally linked with the stomach position—provokes ambiguous interpretation of the phenomenon. 

## 4. Discussion

The cat’s skeleton, found in a burial chamber dating back to between the end of the 6th century and the first half of the 7th century, is an important finding in terms of the illustration of pet–human relationships occurring in the Early to Middle Byzantine period [33]. The fact that the cat—who spent its lifetime living around the feet of the human owner—was laying nearby to the thigh of apparently that same human individual within his/her grave, should be interpreted as a continuation of the inseparable union of these life companions. Observing the status of the burials in “2015-grave-14” chamber, it can be seen that the cat skeleton was carefully placed next to the right femur of the human individual, lying so that the head is pointed towards the west and the feet are pointed towards the east. The inhumation procedural accuracy in both cases (human and animal) seems to be fairly evident proof of the deliberate character of this intentional human activity. Moreover, the cat was placed in the same direction as that of the human corpse, showing evident signs of a bond and an inseparable human–cat relationship. 

Although the Romans were not deeply connected to domestic cats for various reasons [10], it is well known that domestic cats became common in Roman settlements by 100 BC [6,45]. Perhaps the status of cats as “pest controllers” of the households [5] was established by their function as rodent hunters. For this reason, the word “domestic cat” was not found in the important collection of agricultural lore Geoponica (6th century AD), nor was it mentioned by Columella (1st century AD), the prominent writer on agriculture in Roman times [5]. However, the relationship between human and domestic cats was present in far older periods [10]. In particular, the relationship gained more importance due to humans’ preference to accept cats as pet animals since the 2nd to 5th century AD [6]. This new relationship contributed to the widespread acceptance of cats as pet animals in the human household, beyond being simple domestic animals. In this way, cats were eventually able to enter into humans’ private sphere [9]. The human–cat relationship during the Roman period remained in the time periods that followed. In addition to their status as pet animals, they continued to fulfill their role as rodent controllers during the Byzantine period [26]. 

Alongside of being a domestic cat, the Balatlar cat probably played an active role in pest control as well. The cat was carefully placed in the human burial chamber, which is an indication of the status of the animal as a pet. The fact that the cat had captured and killed, or even eaten, a rat just before its death, could be a direct indicator of the cat’s status as a rodent hunter. Moreover, the inhumation of the animal and human individual within the same grave in such a way as described above, provokes us to think that the cat must have been loved and adored by the human individual as a pet animal, beyond that of just being a simple domestic cat. This cat accompanied the human not only here, but also in the afterlife. Additionally, the cat probably hunted rodents as a continuation of its habit from the domestication process. On the other hand, birds were also kept as pet animals in the Roman times and since cats are alleged to be the killers of wild birds [10], this could represent another possible reason as to why there was a lack of preference for cats during the Roman times. 

Although there was a scarcity of cat remains at archaeological sites dating back to the Roman period in Anatolia, for example, Didyma [20]; Pergamon [21]; Lidar Höyük [22]; Pessinus [23]; Troy [24] and Sagalassos [5], a very large assemblage of cat remains from the Yenikapi excavation site demonstrated that cats were significant in the life of the Byzantine capital city Constantinople [27,29,30]. This is an indication that the negative image of the cat during the Roman period [25] was gradually changing in the early Byzantine period as cats started to occupy a place in the social life of the Byzantine world. The context of being buried in the human grave, indeed, illustrates that the Balatlar cat had a comparatively higher status compared to other animals. Although the rat in cat’s stomach could be a good indication of its active role in killing rodents in the household, the sparrow remains could suggest that the cat also ate and hunted wild species such as birds from outside. However, the lack evidence of mastication processes on the bone surfaces could undermine this hypothesis. However, this cat’s hunting habit probably did not affect its status and affectionate relationship at home, since it would appear that it was buried in the very same grave as its possible owner. 

Considering that pet animals can often blur the boundaries between animal and human status, and by being pampered can be treated like other human members of certain households [9], the Balatlar cat appeared to live in the household within these limits. Despite its special status in the household, the hunting habit of both rats and birds was probably an advantage to the household of keeping the cat. Careful examination of the skeletal remains also suggests that the mentioned cat is a normally grown, regular sized house animal, without any health or nutritional deficiency. In addition to the fact that the burial chamber had multiple corpses buried within it, and that the cat was buried next to the right leg of the individual who was buried, the latter suggests that the person was apparently the owner of the cat. Perhaps the good health status of the cat was also affected by the care it received from its owner. The fact that the cat was carefully placed in the same grave as its possible owner could provide proof of the close emotional bond [9] between them. It is also possible that the cat emphasized the social position of its owner and was placed in the grave as an indicator of the owner’s status; a phenomenon often found in Medieval societies and cultures [9]. The most prominent example of this kind is the Byzantine Empress Zoe (11th century) and the cat that was most favorite pet [25]. During the time of the early medieval period, this was an extraordinary example of the status given to a pet animal. 

The morphometrics of the skeletal remains revealed the Balatlar cat as a thin and slender domestic cat. In addition to being a young individual, its gender also probably had an effect on the morphological features of the skeleton. The status of being a young female individual probably also affected its visual morphological characteristics. Its osteometric measurements were very close to the values that Kratochvíl (1973, 1976) [37,38] and Teichert (1978) [39] established as being normal for female cats. In particular, when looking at the Z-score values for the “greatest length” measurements of the Balatlar cat, it was observed that these are almost the same in tone and level with the Z-scores obtained by using the raw data of domestic cats presented by Kratochvíl (1973, 1976) [37,38]. However, the Z-score of the Balatlar cat was different from the Z-scores of the Angora cat and the Roman house cat [36]. This was probably because it was a young female individual. The Balatlar cat was found to be smaller than the Roman house cat examined by von den Driesch and Boessneck (1983) [36], but this was probably because the Roman cat was a male individual. The examined cat also appeared to be a rather small individual when compared to the measurements of wild cats [36,39], since there were notable differences between their Z-scores. On the other hand, although it was a young individual, the animal exhibited characteristics of the final stage of morphological development, according to the times of the epiphyseal closure [44]. 

Moreover, the only explanation of the existence of a beloved cat and human (owner) in the same grave is that their death must have occurred simultaneously, or that the animal was killed intentionally. Given that there is no evidence that the cat was killed, it is also possible that following its natural death the cat was placed in its owner’s grave. The ritual character of inhumation procedures and the existence of the offerings/prey status of the accompanying creatures (rat and sparrow) in the grave could also be potentially explained as the results of a multicultural fusion phenomenon, which occurred in the Roman Empire via the mixing of various traditions, religions, and cultures. It must be added, that such a remnant of the old pagan tradition was still alive in Slavic countries even in the 19th century, therefore, it is not unlikely for the period of Christianized Byzantine Empire that emerged from the Ancient World of polytheism. 

## 5. Conclusions

In conclusion, the Balatlar cat illustrates that the status of cats reached a higher level in the Byzantine society than that in the Roman times. In addition to being a pet animal, it is also an unavoidable fact that the cat also engaged with hunting rodents and birds. The most important signature demonstrating the higher status of human–animal relationship is that the healthy female cat was synchronously placed in the same grave and in the same position as her possible human owner. This can be regarded as the most important example of a pet–human relationship recorded at any Byzantine site so far. 

## Figures and Tables

**Table 1:** Description of the morphometric measurements used in the study.

***Mandibula***		
**No.**	**Measurement**	**Reference**
1	Total length: length from the condyle process-infradentale	von den Driesch, 1976: No.1 [35]; von den Driesch and Boessneck, 1983: No.1 [36]; Kratochvil, 1973: No.2 [37]; Teichert, 1978: No.2 [39]; Guintard and Arnaud, 2003: No.1 [40]
2	Length from the indentation between the condyle process and the angular process-infradentale	von den Driesch, 1976: No.2 [35]; von den Driesch and Boessneck, 1983: No.3 [36]; Kratochvil, 1973: No.4 [37]; Teichert, 1978: No.4 [39]; Guintard and Arnaud, 2003: No.2 [40]
3	The condyle process-aboral border of the canine alveolus	von den Driesch, 1976: No.3 [35]; von den Driesch and Boessneck, 1983: No.5 [36]; Kratochvil, 1973: No.5 [37]; Teichert, 1978: No.5 [39]; Guintard and Arnaud, 2003: No.3 [40]
4	Length from the indentation between the condyle process and the angular process-aboral border of the canine alveolus	von den Driesch, 1976:No.4 [35]; Kratochvil, 1973: No.6 [37]; Teichert, 1978: No.6 [39]; Guintard and Arnaud, 2003: No.4 [40]
5	Length from the angular process-infradentale	von den Driesch and Boessneck, 1983: No.2 [36]; Kratochvil, 1973: No.3 [37]; Teichert, 1978: No.3 [39]; Guintard and Arnaud, 2003: No.11 [40]
6	Length from the condyle process-oral border of the canine alveolus	von den Driesch and Boessneck, 1983: No.4 [36]
7	Length from the coronoid process–infradentale	Kratochvil, 1973: No.1 [37]; Teichert, 1978: No.4 [39]
8	Length from the angular process-aboral border of the canine alveolus	Kratochvil, 1973: No.7 [37]; Teichert, 1978: No.7 [39]
9	Length of the cheektooth row, P3-M1, measured along the alveoli	von den Driesch, 1976:No.5 [35]; von den Driesch and Boessneck, 1983: No.9 [36]; Kratochvil, 1973: No.9 [37]; Teichert, 1978: No.9 [39]; Guintard and Arnaud, 2003: No.5 [40]
10	Length of the carnassial (M1), measured at the cingulum	von den Driesch, 1976: No.6-length [35]; von den Driesch and Boessneck, 1983: No.10-length [36]; Kratochvil, 1973: No.11 [37]; Teichert, 1978: No.11 [39]; Guintard and Arnaud, 2003: No.6 [40]
11	Breadth of the carnassial (M1), measured at the cingulum	von den Driesch, 1976:No.6-breadth [35]; von den Driesch and Boessneck, 1983: No.10-breadth [36]; Kratochvil, 1973: No.12 [37]; Teichert, 1978: No.12 [39]; Guintard and Arnaud, 2003: No.6a [40]
12	Length of the carnassial alveolus	von den Driesch, 1976:No.7 [35]; Guintard and Arnaud, 2003: No.7 [40]
13	Length of the premolar row, P3-P4, measured along the alveoli	Kratochvil, 1973: No.10 [37]; Teichert, 1978: No.10 [39]
14	Height of the vertical ramus (Ramus mandibulae): basal point of the angular process-Coronion	von den Driesch, 1976:No.8 [35]; von den Driesch and Boessneck, 1983: No.11 [36]; Kratochvil, 1973: No.15 [37]; Teichert, 1978: No.15 [39]; Guintard and Arnaud, 2003: No.8 [40]
15	Height of the mandible behind M1, measured on the buccal side	von den Driesch, 1976:No.9 [35]; von den Driesch and Boessneck, 1983: No.12 [36]; Kratochvil, 1973: No.14 [37]; Teichert, 1978: No.14 [39]; Guintard and Arnaud, 2003: No.9 [40]
16	Height of the mandible between P3-P4, measured on the buccal side	Kratochvil, 1973: No.13 [37]; Teichert, 1978: No.13 [39]
17	Heigth of the mandible in front of P3, measured on the buccal side	von den Driesch, 1976:No.10 [35]; Guintard and Arnaud, 2003: No.10 [40]
18	Length of the C1-M1: length of the aboral border of the canine alveolus-the aboral border of the M1 alveolus	Kratochvil, 1973: No.8 [37]; Teichert, 1978: No.8 [39]
19	Length of the aboral border of the M1 alveolus-infradentale	Guintard and Arnaud, 2003: No.12 [40]
***Scapula***		
**No**	**Measurement**	**Reference**
1	Height along the spine	von den Driesch, 1976: HS [35]; von den Driesch and Boessneck, 1983: HS [36]; Kratochvil, 1976: No.11 [38]
2	Diagonal height: From the most distal point of the scapula to the thoracic angle	von den Driesch, 1976: DHA [35]; von den Driesch and Boessneck, 1983: DHA [36]
3	Greatest dorsal length	von den Driesch, 1976: Ld [35]; Kratochvil, 1976: No.12 [38]
4	Smallest length of the collum scapulae	von den Driesch, 1976: SLC [35]; von den Driesch and Boessneck, 1983: SLC [36]; Kratochvil, 1976: No.13 [38]
5	Greatest length of the processus articularis (glenoid)	von den Driesch, 1976: GLP [35]; von den Driesch and Boessneck, 1983: GLP [36]; Kratochvil, 1976: No.14 [38]
6	Length of the glenoid cavity	von den Driesch, 1976: LG [35]; von den Driesch and Boessneck, 1983: LG [36]; Kratochvil, 1976: No.15 [38]
7	Breadth of the glenoid cavity	von den Driesch, 1976: BG [35]; von den Driesch and Boessneck, 1983: BG [36]; Kratochvil, 1976: No.16 [38]
8	Height in the area of the spina scapulae	Kratochvil, 1976: No.10 [38]
***Humerus***		
**No**	**Measurement**	**Reference**
1	Greatest length	von den Driesch, 1976: GL [35]; von den Driesch and Boessneck, 1983: GL [36]; Kratochvil, 1976: No.17 [38]
2	Greatest length from caput	von den Driesch, 1976: GLC [35]; von den Driesch and Boessneck, 1983: GLC [36]
3	Greatest breadth of the proximal end	von den Driesch, 1976: Bp [35]; von den Driesch and Boessneck, 1983: Bp [36]
4	Depth of the proximal end	von den Driesch, 1976: Dp [35]; von den Driesch and Boessneck, 1983: Dp [36]; Kratochvil, 1976: No.18 [38]
5	Smallest breadth of the diaphysis	von den Driesch, 1976: SD [35]; von den Driesch and Boessneck, 1983: SD [36]; Kratochvil, 1976: No.19 [38]
6	Breadth of the distal end	von den Driesch, 1976: Bd [35]; von den Driesch and Boessneck, 1983: Bd [36]; Kratochvil, 1976: No.20 [38]
***Radius***		
**No**	**Measurement**	**Reference**
1	Greatest length	von den Driesch, 1976: GL [35]; von den Driesch and Boessneck, 1983: GL [36]; Kratochvil, 1976: No.21 [38]
2	Greatest breadth of the proximal end	von den Driesch, 1976: Bp [35]; von den Driesch and Boessneck, 1983: Bp [36]; Kratochvil, 1976: No.22 [38]
3	Depth of the proximal end	Kratochvil, 1976: No.23 [38]
4	Smallest breadth of the diaphysis	von den Driesch, 1976: SD [35]; von den Driesch and Boessneck, 1983: SD [36]; Kratochvil, 1976: No.24 [38]
5	Greatest breadth of the distal end	von den Driesch, 1976: Bd [35]; von den Driesch and Boessneck, 1983: Bd [36]; Kratochvil, 1976: No.25 [38]
***Ulna***		
**No**	**Measurement**	**Reference**
1	Greatest length	von den Driesch, 1976: GL [35]; von den Driesch and Boessneck, 1983: GL [36]; Kratochvil, 1976: No.26 [38]
2	Length of the olecranon	von den Driesch, 1976: LO [35]
3	Depth across the processus anconeus	von den Driesch, 1976: DPA [35]; von den Driesch and Boessneck, 1983: DPA [36]; Kratochvil, 1976: No.27 [38]
4	Smallest depth of the olecranon	von den Driesch, 1976: SDO [35]; von den Driesch and Boessneck, 1983: SDO [36]
5	Breadth across the coronoid process	von den Driesch, 1976: BPC [35]; von den Driesch and Boessneck, 1983: BPC [36]; Kratochvil, 1976: No.28 [38]
***Ossa coxae***		
**No**	**Measurement**	**Reference**
1	Greatest length of one half	von den Driesch, 1976: GL [35]; von den Driesch and Boessneck, 1983: GL [36]; Kratochvil, 1976: No.39 [38]
2	Greatest length of acetabulum including the lip	von den Driesch, 1976: LA [35]
3	Greatest length of acetabulum on the rim	von den Driesch, 1976: LAR [35]; von den Driesch and Boessneck, 1983: LAR [36]; Kratochvil, 1976: No.43 [38]
4	Length of the symphysis	von den Driesch, 1976: LS [35]; Kratochvil, 1976: No.42 [38]
5	Smallest height of the shaft of ilium	von den Driesch, 1976: SH [35]; Kratochvil, 1976: No.45 [38]
6	Smallest breadth of the shaft of ilium	von den Driesch, 1976: SB [35]; Kratochvil, 1976: No.46 [38]
7	Inner length of the foramen obturatum	von den Driesch, 1976: Lfo [35]
***Femur***		
**No**	**Measurement**	**Reference**
1	Greatest length	von den Driesch, 1976: GL [35]; von den Driesch and Boessneck, 1983: GL [36]; Kratochvil, 1976: No.51 [38]
2	Greatest breadth of the proximal length	von den Driesch, 1976: Bp [35]; von den Driesch and Boessneck, 1983: Bp [36]; Kratochvil, 1976: No.52 [38]
3	Greatest depth of the caput femoris	von den Driesch, 1976: DC [35]; Kratochvil, 1976: No.53 [38]
4	Smallest breadth of the diaphysis	von den Driesch, 1976: SD [35]; von den Driesch and Boessneck, 1983: SD [36]; Kratochvil, 1976: No.54 [38]
5	Greatest breadth of the distal end	von den Driesch, 1976: Bd [35]; von den Driesch and Boessneck, 1983: Bd [36]; Kratochvil, 1976: No.55 [38]
***Tibia***		
**No**	**Measurement**	**Reference**
1	Greatest length	von den Driesch, 1976: GL [35]; von den Driesch and Boessneck, 1983: GL [36]; Kratochvil, 1976: No.58 [38]
2	Greatest breadth of the proximal end	von den Driesch, 1976: Bp [35]; von den Driesch and Boessneck, 1983: Bp [36]; Kratochvil, 1976: No.59 [38]
3	Smallest breadth of the diaphysis	von den Driesch, 1976: SD [35]; von den Driesch and Boessneck, 1983: SD [36]; Kratochvil, 1976: No.60 [38]
4	Greatest breadth of the distal end	von den Driesch, 1976: Bd [35]; von den Driesch and Boessneck, 1983: Bd [36]
5	Greatest depth of the distal end	von den Driesch, 1976: Dd [35]; Kratochvil, 1976: No.61 [38]
***Fibula***		
**No**	**Measurement**	**Reference**
1	Greatest length	von den Driesch, 1976: GL [35]; von den Driesch and Boessneck, 1983: GL [36]; Kratochvil, 1976: No.62 [38]
***Talus***		
**No**	**Measurement**	**Reference**
1	Greatest length	von den Driesch, 1976: GL [35]; Kratochvil, 1976: No.63 [38]
***Calcaneus***		
**No**	**Measurement**	**Reference**
1	Greatest length	von den Driesch, 1976: GL [35]; von den Driesch and Boessneck, 1983: GL [36]; Kratochvil, 1976: No.64 [38]
2	Greatest breadth	von den Driesch, 1976: GB [35]; von den Driesch and Boessneck, 1983: GB [36]; Kratochvil, 1976: No.65 [38]
***Metapodials***		
**No**	**Measurement**	**Reference**
1	Greatest length	von den Driesch, 1976: GL [35]; von den Driesch and Boessneck, 1983: GL [36]; Kratochvil, 1976: No.29, 30, 32, 34, 36, 66, 68, 70, 72 [38]
2	Greatest breadth of the distal end	von den Driesch, 1976: Bd [35]; von den Driesch and Boessneck, 1983: Bd [36]; Kratochvil, 1976: No.31, 33, 35, 67, 69, 71, 73 [38]
***Atlas***		
**No**	**Measurement**	**Reference**
1	Greatest breadth over the wings	von den Driesch, 1976: GB [35]; von den Driesch and Boessneck, 1983: GB [36]; Kratochvil, 1976: No.4 [38]
2	Greatest length	von den Driesch, 1976: GL [35]; von den Driesch and Boessneck, 1983: GL [36]; Kratochvil, 1976: No.1 [38]
3	Greatest breadth of the facies articularis cranialis	von den Driesch, 1976: BFcr [35]; von den Driesch and Boessneck, 1983: BFcr [36]; Kratochvil, 1976: No.2 [38]
4	Greatest breadth of the facies articularis caudalis	von den Driesch, 1976: BFcd [35]; von den Driesch and Boessneck, 1983: BFcd [36]; Kratochvil, 1976: No.3 [38]
5	Greatest length from the facies articularis cranialis to the facies articularis caudalis	von den Driesch, 1976: GLF [35]; von den Driesch and Boessneck, 1983: GLF [36]
6	Length of the arcus dorsalis	von den Driesch, 1976: Lad [35]
7	Height	von den Driesch, 1976: H [35]
***Axis***		
**No**	**Measurement**	**Reference**
1	Greatest length in the region of the corpus including dens	von den Driesch, 1976: LCDe [35]; von den Driesch and Boessneck, 1983: LCDe [36]; Kratochvil, 1976: No.5 [38]
2	Greatest length of the arch including the processus articulares caudales	von den Driesch, 1976: LAPa [35]; von den Driesch and Boessneck, 1983: LAPa [36]
3	Greatest breadth of the facies articularis cranialis	von den Driesch, 1976: BFcr [35]; von den Driesch and Boessneck, 1983: BFcr [36]; Kratochvil, 1976: No.6 [38]
4	Greatest breadth across the processus articulares caudales	von den Driesch, 1976: BPacd [35]; von den Driesch and Boessneck, 1983: BPacd [36]
5	Greatest breadth across the processus transversi	von den Driesch, 1976: BPtr [35]
6	Smallest breadth of the vertebra	von den Driesch, 1976: SBV [35]; von den Driesch and Boessneck, 1983: SBV [36]; Kratochvil, 1976: No.7 [38]
7	Greatest breadth of the facies articularis caudalis	von den Driesch, 1976: BFcd [35]
8	Greatest height	von den Driesch, 1976: H [35]; von den Driesch and Boessneck, 1983: H [36]; Kratochvil, 1976: No.8 [38]
9	Height of the processus spinosus cranially	Kratochvil, 1976: No.9 [38]
***Sacrum***		
**No**	**Measurement**	**Reference**
1	Greatest length on the ventral side	von den Driesch, 1976: GL [35]; von den Driesch and Boessneck, 1983: GL [36]
2	Physiological length	von den Driesch, 1976: PL [35]; Kratochvil, 1976: No.37 [38]
3	Greatest breadth across the wings	von den Driesch, 1976: GB [35]; von den Driesch and Boessneck, 1983: GB [36]; Kratochvil, 1976: No.38 [38]
4	Greatest breadth of the facies articularis cranialis	von den Driesch, 1976: BFcr [35]
5	Greatest height of the facies articularis cranialis	von den Driesch, 1976: HFcr [35]

**Table 2:** Calculated age of the Balatlar cat using the time index of epiphyseal closure [44].

Bone	Epiphyseal Plate Closure	Smith 1969		Balatlar Cat
		DAYS	MONTH	Fused/unfused
Humerus	Medial condyle	98	3.3	Fused
Humerus	Lateral condyle	98	3.3	Fused
Scapula	Tuber&corocoid proccess	112	3.7	Fused
Humerus	Medial epicondyle	112–126	3.7–4.2	Fused
Radius	Proximal	196	6.5	Fused
Femur	Greater trochanter	196–232	6.5–7.7	Fused
Metacarpus	Distal epiphysis II–V	203–280	6.8–9.3	Fused
Femur	Femoral head	210–280	7–9.3	Unfused
Metatarsus	Distal epiphysis II–V	224–308	7.5–10.3	Fused
Femur	Lesser trochanter	238–308	7.9–10.3	Fused
Ulna	Proximal (tuberosity of olecranon	266–364	8.9–12.1	Fused
Tibia	Distal epiphysis	280–364	9.3–12.1	Fused
Fibula	Distal epiphysis	280–392	9.3–13	Fused
Tibia	Proximal epiphysis	350–532	11.7–17.7	Unfused
Tibia	Tibial tuberosity	350–532	11.7–17.7	Unfused
Fibula	Proximal epiphysis	378–504	12.6–16.8	Unfused
Femur	Distal epiphysis	379–532	12.6–17.7	Unfused
Radius	Distal	406–616	13.5–20.5	Unfused
Ulna	Distal epiphysis	406–700	13.5–23.3	Unfused
Humerus	Proximal epiphysis	547–730	18.2–24.3	Unfused

**Table 3:** The mandible measurements of the Balatlar cat in comparison with literature data.

Measure Number	Bodyside	Balatlar Cat	Angora Cat *	von den Driesch and Boessneck 1983, *Felis silvestris f. catus*	Kratochvil 1973, *Felis silvestris f. catus*	Kratochvil 1973, *Felis s. silvestris*	Teichert 1978, *Felis silvestris f. catus*	Teichert 1978, *Felis silvestris silvestris*	Guintard and Arnaud 2003, *Felis catus*
1	Left	55.01	63.55	72.00	57.27	66.34	61.40	63.70	58.50
	Right	54.52	62.31						
Total length (no:1)	Z-score (F.C)	−1.292	1.216		−0.524		0.746		−0.146
	Z-score (F.S)	−1.127				0.781		0.345	
2	Left	51.52	57.91	67.00	53.43	62.65	59.40	62.20	55.80
	Right	51.61	58.30						
3	Left	48.37	56.38	64.00	50.31	58.21	54.40	55.80	51.30
	Right	48.51	57.04						
4	Left	44.96	51.25		46.63	54.63	52.10	54.50	49.10
	Right	45.33	51.50						
5	Left	53.34	60.26	69.00	55.48	65.46	61.60	65.20	57.20
	Right	53.51	60.35						
6	Left	52.80	60.81	69.00					
	Right	52.84	60.44						
7	Left	54.06	60.40		58.31	66.10	64.10	64.70	
	Right	53.99	60.95						
8	Left	53.33	53.78		48.74	57.51	54.30	57.60	
	Right	53.53	53.61						
9	Left	18.89	19.59	21.00	18.41	21.70	20.00	21.20	18.90
	Right	18.96	19.22						
10	Left	7.16	6.23	8.00	7.00	8.55	7.30	8.50	7.20
	Right	7.15	6.31						
11	Left	3.18	3.12	3.00	3.20	3.73	3.10	3.60	3.10
	Right	3.20	3.19						
12	Left	7.78	7.02	64.00					7.00
	Right	8.19	7.19						
13	Left	11.89	12.67		11.29	13.39	12.50	13.00	
	Right	11.93	12.78						
14	Left	23.76	27.25	29.00	23.54	28.32	25.50	28.50	25.00
	Right	23.75	27.82						
15	Left	9.88	10.66	12.00	10.10	11.89	11.40	11.40	9.80
	Right	9.78	10.25						
16	Left	8.51	10.45		9.38	11.15	10.30	10.70	
	Right	8.39	10.02						
17	Left	8.86	10.28						9.60
	Right	8.93	10.37						
18	Left	23.85	26.05		25.00	28.58	27.80	27.90	
	Right	23.84	26.05						
19	Left	30.28	32.23						32.60
	Right	30.30	32.65						

* Reference collection; F.C: *Felis silvestris f. catus*; F.S: *Felis silvestris silvestris*.

**Table 4:** The scapula measurements of the Balatlar cat in comparison with literature data.

SCAPULA	Bodyside	1	Height Along the Spine (n.1), Z-Score	2	3	4	5	6	7	10
BALATLAR CAT	Left	64.06	−0.978	63.27	44.64	10.86	11.97	10.89	7.89	58.34
	Right	64.15		63.58	44.75	10.90	12.07	10.95	7.76	58.41
ANGORA CAT *	Left	79.41	0.876	77.28	55.35	13.54	15.02	13.23	10.05	73.07
	Right	79.56		77.39	55.27	13.65	15.10	13.23	10.01	73.20
von den Driesch and Boessneck 1983, *Felis silvestris f. catus*	Left	79.00	0.848	82.00		13.70	16.50	15.00	10.70	
	Right	79.50		82.50		13.50	16.00	14.50	10.50	
Kratochvil 1976, *Felis silvestris f. catus ♀*	Mean	66.02	−0.747		48.70	11.61	13.16	11.06	8.69	61.23

* Reference collection.

**Table 5:** The humerus measurements of the Balatlar cat in comparison with literature data.

Humerus	Bodyside	1	Greatest Length (no.1), Z-Score	3	4	5	6	7
BALATLAR CAT	Left	92.96	−0.785	92.38	14.11	18.44	5.55	15.73
	Right	93.02		92.40	14.71	18.05	5.59	15.63
ANGORA CAT *	Left	101.77	0.075	100.46	16.87	20.73	8.34	20.02
	Right	102.12		100.52	17.06	20.37	8.17	19.68
von den Driesch and Boessneck 1983, *Felis silvestris f. catus*	Left	115.50	1.386	114.00		24.80	8.80	20.20
	Right	115.70		114.00		24.50	9.00	20.30
Kratochvil 1976, *Felis silvestris f. catus* ♀	Mean	94.13	−0.676			19.66	6.33	17.34

* Reference collection.

**Table 6:** The radius and ulna measurements of the Balatlar cat in comparison with literature data.

	Measurements>	Body Side	1	Greatest Length (no.1), Z-Score	2	3	4	5
Radius	BALATLAR CAT	Left	88.74	−0.826	6.87	5.35	5.48	11.44
		Right	89.86		6.84	5.32	5.26	11.43
	ANGORA CAT *	Left	99.57	0.429	8.00	6.05	6.43	12.84
		Right	99.89		8.24	6.04	6.41	12.93
	von den Driesch and Boessneck 1983, *Felis silvestris f. catus*	Left	107.50	1.212	9.60		6.00	13.80
		Right	105.00		9.50		6.30	14.20
	Kratochvil 1976, *Felis silvestris f. catus*♀	Mean	89.38	−0.816	7.70	5.60	5.03	12.00
Ulna	BALATLAR CAT	Left	104.10	0.316	9.60	10.55	9.29	8.55
		Right	105.29		10.89	10.23	9.25	8.56
	ANGORA CAT *	Left	115.69	0.785	11.14	12.11	10.57	9.85
		Right	115.95		11.42	11.98	10.59	9.78
	von den Driesch and Boessneck 1983, *Felis silvestris f. catus*	Left		−1.466		13.50		11.00
		Right	125.00			13.00	11.70	10.50
	Kratochvil 1976, *Felis silvestris f. catus*♀	Mean	105.88	0.366		10.41		8.37

* Reference collection.

**Table 7:** The ossa coxae measurements of the Balatlar cat and its comparison with literature data.

COXAE	Bodyside	1	Greatest Length of One Half (No.1), Z-Score	2	3	4	5	6	7
BALATLAR CAT	Left		−1.129						
	Right	72.25		10.74	9.44		9.58	4.18	18.96
ANGORA CAT *	Left	84.38	0.692	13.80	11.12	34.86	12.68	5.72	21.07
	Right	83.91		12.72	11.06	34.62	12.76	5.34	21.33
von den Driesch and Boessneck 1983, *Felis silvestris f. catus*	Left	86.00	0.977		14.00				
	Right	86.00			14.00				
Kratochvil 1976, *Felis silvestris f. catus* ♀	Mean	76.10	−0.540		10.51	27.14	10.42	4.35	

* Reference collection.

**Table 8:** The femur measurements of the Balatlar cat in comparison with literature data.

Femur	Bodyside	1	Greatest Length (no.1), Z-Score	2	3	4	5
BALATLAR CAT	Left	101.71	−0.791	18.29	8.76	7.38	16.89
	Right	102.16		18.57	8.85	7.41	17.20
ANGORA CAT *	Left	112.34	0.169	21.01	10.38	9.95	19.02
	Right	113.14		21.02	10.39	10.16	19.11
von den Driesch and Boessneck 1983, *Felis silvestris f. catus*	Left	126.50	1.348	23.00		11.00	22.00
	Right	125.50		22.80		10.50	21.00
Kratochvil 1976, *Felis silvestris f. catus*♀	Mean	102.66	−0.727	19.51	9.48	7.99	17.84

* Reference collection.

**Table 9:** The tibia measurements of the Balatlar cat in comparison with literature data.

Tibia	Bodyside	1	Greatest Length (no.1), Z-Score	2	3	4	5
BALATLAR CAT	Left	109.30	−0.740	17.94	6.84	12.92	8.99
	Right	109.27		17.94	6.66	13.45	8.95
ANGORA CAT *	Left	117.87	0.209	21.04	8.36	14.89	10.01
	Right	116.72		20.98	8.48	14.65	
von den Driesch and Boessneck 1983, *Felis silvestris f. catus*	Left	126.50	1.330	22.50	9.50	16.00	
	Right	127.00		22.50	9.50	16.00	
Kratochvil 1976, *Felis silvestris f. catus* ♀	Mean	108.78	−0.800	18.78	6.92		9.17

* Reference collection.

**Table 10:** The fibula measurements of the Balatlar cat in comparison with literature data.

Fibula		1	Greatest Length (no.1), Z-Score
BALATLAR CAT	Left	101.93	−0.771
	Right	101.17	
ANGORA CAT *	Left	110.63	0.283
	Right	110.99	
von den Driesch and Boessneck 1983, *Felis silvestris f. catus*	Left	119.7	1.294
	Right		
Kratochvil 1976, *Felis silvestris f. catus* ♀	Mean	101.24	−0.806

* Reference collection.

**Table 11:** The astragalus and calcaneus measurements of the Balatlar cat in comparison with literature data.

Bone>		AST	Greatest Length (no.1)	CAL	Greatest Length (no.1)	CAL
Measurements>		1	Z-Score	1	Z-Score	2
BALAT CAT	Left	14.42	−1.040	26.4	−1.194	10.88
	Right	14.44		26.57		11.8
ANGORA CAT *	Left	16.28	0.954	30.29	0.588	12.63
	Right	16.12		30.33		12.71
von den Driesch and Boessneck 1983, *Felis silvestris f. catus*	Left			31.00	1.026	13.30
	Right			31.50		14.00
Kratochvil 1976, *Felis silvestris f. catus* ♀	Mean	15.43	0.086	28.15	−0.420	11.57

* Reference collection: AST: Astragalus; CAL: Calcaneus.

**Table 12:** The metapodials measurements of the Balatlar cat in comparison with literature data.

Metapodial Bone Number>				1	2	3	4	5
BALATLAR CAT	Metacarpus	Left	GL		26.80	30.77	28.94	23.87
			Bd		4.55	4.79	4.26	4.40
		Right	GL		26.92	30.92	29.14	24.28
			Bd		4.49	4.85	4.31	4.31
	Metatarsus	Left	GL		44.34	48.97	48.33	43.26
			Bd		4.65	5.98	5.77	5.00
		Right	GL		44.88	48.43	49.17	43.62
			Bd		4.71	5.68	5.28	4.80
ANGORA CAT *	Metacarpus	Left	GL		30.14	34.72	33.45	27.66
			Bd		4.85	5.19	4.76	4.73
		Right	GL		30.39	34.95	33.57	27.92
			Bd		5.32	5.2	4.77	4.72
	Metatarsus	Left	GL		51.11	53.47	54.47	47.43
			Bd		5.2	6.32	5.73	5.89
		Right	GL		51.16	53.34	54.41	47.17
			Bd		5.04	6.18	5.58	5.63
von den Driesch and Boessneck 1983, *Felis silvestris f. Catus*	Metacarpus		GL	13.00	33.70	38.30	36.70	31.30
			Bd		5.30	5.80	5.50	4.90
	Metatarsus		GL	53.30	53.30	58.80	58.50	56.30
			Bd	5.70	5.80	7.00	6.20	5.00
Kratochvil 1976, *Felis silvestris f. catus* ♀	Metacarpus		GL	10.77	27.34	31.45	29.91	24.99
			Bd		4.24	4.36	4.09	
	Metatarsus		GL		43.75	48.09	48.80	46.70
			Bd		4.72	5.17	4.77	4.32

* Reference collection. GL: greatest length.

**Table 13:** The atlas, epistropheus and sacrum measurements of the Balatlar cat in comparison with literature data.

ATLAS	1	Z-Score (No.1)	2	3	4	5	6	7		
BALATLAR CAT	30.08	−1.057	18.98	21.35	15.18	16.42	8.50	13.24		
ANGORA CAT *	37.11	0.396	21.15	23.81	16.97	17.06	8.59	14.42		
von den Driesch and Boessneck 1983, *Felis silvestris f. catus*	41.00	1.199	23.30	25.00	18.50	20.00				
Kratochvil 1976, *Felis silvestris f. catus*♀	32.59	−0.538	16.62	21.32	15.01					
EPISTROPHEUS	1	Z-score (No.1)	2	3	4	5	6	7	8	9
BALATLAR CAT	22.67	−0.761	23.01	14.64	14.98		9.27	8.97	18.54	6.45
ANGORA CAT *	25.38	0.146	28.78	15.90	18.19	16.94	10.30	10.32	21.13	7.41
von den Driesch and Boessneck 1983, *Felis silvestris f. catus*	29.00	1.359	29.00	17.00	17.50		11.50			
Kratochvil 1976, *Felis silvestris f. catus*♀	22.72	−0.744		15.13			11.11		19.16	7.16
SACRUM	1	Z-score (No.1)	2	3	4	5				
BALATLAR CAT	26.66	−1.153	22.98	27.11	12.55	5.00				
ANGORA CAT *	32.63	0.524	26.26	29.15	14.09	6.26				
von den Driesch and Boessneck 1983, *Felis silvestris f. catus*	33.00	0.630		28.00						
Kratochvil 1976, *Felis silvestris f. catus*♀			24.40	27.70						

* Reference collection.

**Table 14:** The long bone indices of the Balatlar cat in comparison with literature data.

CAT		Humerus Index		HI Z-Score	Radius Index		RI Z-Score	Femur Index		FI Z-Score	Tibia Index		TI Z-Score
BALATLAR CAT	Left	5.97	5.99	−1.196	6.18	6.02	0.136	7.26	7.26	−1.153	6.26	6.18	−1.006
	Right	6.01			5.85			7.25			6.09		
ANGORA CAT *	Left	8.19	8.10	1.018	6.46	6.44	1.341	8.86	8.92	1.066	7.09	7.18	0.584
	Right	8.00			6.42			8.98			7.27		
von den Driesch and Boessneck 1983, *Felis silvestris f. catus*	Left	7.62	7.70	0.598	5.58	5.79	−0.523	8.70	8.54	0.558	7.51	7.50	1.093
	Right	7.78			6.00			8.37			7.48		
Kratochvil 1976, *Felis silvestris f. catus* ♀	*Mean*		6.73	−0.420		5.64	−0.954		7.77	−0.471		6.39	−0.672

* Reference collection; HI: Humerus index; RI: Radius index; FI: Femur index; TI: Tibia index.

## Data Availability

The Balatlar cat and the Angora cat are available in the collections of the Osteoarchaeology Research Center, Istanbul University-Cerrahpaşa. Skeletal remains of the Balatlar cat are available for further study via application through the General Directorate of Cultural Heritage and Museums, Ministry of Culture and Tourism, Republic of Turkey. The datasets generated, analyzed, and used in this study will be provided by the corresponding author on request.
